# Reply to Taal et al. Comment on “Iasella et al. Close Follow-Up of Patients with Neurofibromatosis Type 1 Reduces the Incidence of Malignant Peripheral Nerve Sheath Tumour. *Cancers* 2025, *17*, 1306”

**DOI:** 10.3390/cancers17203356

**Published:** 2025-10-17

**Authors:** Maria Pia Iasella, Dries Ruttens, Daphne Hompes, Vincent Vandecaveye, Raf Sciot, Christophe Deroose, Thomas Douchy, Thomas Decramer, Sandra Jacobs, Ellen Denayer, Frank Van Calenbergh, Eric Legius, Hilde Brems

**Affiliations:** 1 Centre for Human Genetics, University Hospitals Leuven, KU Leuven, 3000 Leuven, Belgiumeric.legius@kuleuven.be (E.L.); 2 Department of Paediatric Oncology, University Hospitals Leuven, 3000 Leuven, Belgium; 3 Department of Paediatric Oncology, Princess Máxima Centre for Pediatric Oncology, 3584 CS Utrecht, The Netherlands; 4 Department of Surgical Oncology, University Hospitals Leuven, 3000 Leuven, Belgium; daphne.hompes@uzleuven.be (D.H.);; 5 Radiology Department, University Hospitals Leuven, 3000 Leuven, Belgium; 6 Department of Pathology, University Hospitals Leuven, KU Leuven, 3000 Leuven, Belgium; 7 Nuclear Medicine, University Hospitals Leuven, and Nuclear Medicine and Molecular Imaging, Department of Imaging and Pathology, KU Leuven, 3000 Leuven, Belgium; 8 Department of Neurosurgery, University Hospitals Leuven, 3000 Leuven, Belgium

We would like to sincerely thank Dr. Taal and colleagues for their thoughtful and detailed comments on our recent retrospective cohort study in *Cancers* [1]. We are grateful for the opportunity to address the valuable comments and concerns raised.

As acknowledged in our Discussion, we agree with the inherent limitations of a retrospective design. The main message is that our study provides support for the recommendation of close tumour surveillance in adolescents and adults with neurofibromatosis type 1 (NF1). We used whole-body diffusion-weighted magnetic resonance imaging (WB-DW/MRI) as part of our surveillance programme, but its specific role needs to be further studied in prospective studies, for example, studies comparing different imaging modalities as part of a surveillance programme. We believe that collecting and analysing retrospective data is essential before establishing well-designed prospective clinical studies.

Reliance on a historical control group has inherent limitations. However, to our knowledge, the data from Uusitalo et al., 2016, represents the best available epidemiological data for NF1-associated malignancies to date [2]. The incidence of NF1 in the Finnish population is ~1:1900, contrasting with the prevalence of 1:3850 reported in Uusitalo et al., 2016 [2]. This number relates to a lower prevalence in the older age groups, likely resulting both from a high mortality already under 50 years of age and losses to follow-up [3]. The latter supports our central message that consistent and long-term follow-up of adults with NF1 is essential to ensure timely diagnosis and optimal care. In contrast to the Finnish population-based data, our cohort is drawn from a university hospital setting, which may result in bias towards more severely affected patients, as stated in the Discussion of the manuscript. Individuals from our centre have a rather high standardised incidence rate for cancers compared to other centres in Europe, as pointed out in a recent study comparing cancer in NF1 patients both with and without an *NF1*-microdeletion (Kallionpää et al., manuscript in preparation). Consequently, the predicted prevalence of malignant peripheral nerve sheath tumours (MPNSTs) in our patient cohort may be higher than in more general population-based cohorts. To address potential overestimation of the expected MPNST cases, however, we also performed an even more conservative calculation using a 20% lower lifetime MPNST risk (12% instead of 15%), in line with previous studies [4,5]. Even under these assumptions, our findings remained statistically significant (*p* = 0.041), supporting our reported findings.

Regarding the associated burden of surveillance, we agree that the balance between benefit and harm of surveillance is a critical issue. Due to the retrospective nature of our study, we were unable to systematically capture data on patient anxiety or healthcare utilisation burden and costs. Nevertheless, based on our clinical experience, most patients report feeling reassured by being in a structured follow-up programme.

Of the fifteen ANNUBPs (atypical neurofibromatous neoplasms of uncertain biologic potential) that were surgically removed, all fifteen were asymptomatic at the time of diagnosis; in addition, thirteen were detected through WB-DW/MRI, one via follow-up imaging for GIST (gastrointestinal stromal tumour), and one through PET-CT (Positron Emission Tomography/Computed Tomography) prompted by clinical symptoms at another location. While some incidental findings did lead to further investigations or surgeries, the number of “unnecessary” interventions was limited. In total, sixty-five surgeries were performed in fifty-eight individuals (fifteen ANNUBPs, fourteen secretory pheochromocytomas, five GISTs, one brain tumour, twenty-eight benign neurofibromas, one desmoid tumour, and one lymph node consistent with Castleman disease). Of the twenty-eight benign neurofibromas removed, suspicion for malignant transformation was present in only seven. Of these, three fulfilled one out of four histological criteria for ANNUBP [6]. Further molecular analysis in one of these did not show *CDKN2A/2B* deletion. In the remaining cases molecular analysis was not performed, which makes it impossible to rule out the presence of ANNUBP. In the twenty-one remaining cases, surgery was performed for various clinical reasons unrelated to the whole-body MRI findings (thirteen due to functional complaints, three due to pain, three due to esthetical reasons, two due to important haemorrhage in neurofibromas). Of these twenty-one cases, three fulfilled one out of four histological criteria for ANNUBP. No molecular analysis for *CDKN2A/2B* deletion was performed. We diagnosed and treated fourteen cases of secretory pheochromocytoma (of which eleven where asymptomatic and three were symptomatic with arterial hypertension) and five cases of GIST (all asymptomatic). Treatment of pheochromocytoma and GIST before they become symptomatic possibly decreases morbidity and mortality associated with these conditions [7,8]. We had no surgical deaths in our cohort, and severe complications from surgery were not observed.

We completely agree that quality of life (QoL) is a crucial endpoint. Unfortunately, as Dr. Taal and colleagues noted, our retrospective design did not permit a structured assessment of QoL outcomes. We believe it is important to include this aspect in future prospective trials.

To conclude, our findings provide sufficient evidence for the benefit of close surveillance in adolescents and adults with NF1. Based on this evidence we advocate for maintained close surveillance in adult NF1 patients, a group who is at increased risk for loss to follow-up. Future larger, prospective clinical trials are needed in order to confirm our findings and to study the specific role of WB-DW/MRI, PET-MRI, and other imaging modalities as part of a surveillance programme.

We thank Dr. Taal and colleagues again for their insightful feedback and for contributing to constructive dialogue on improving care for NF1 patients.

## Data Availability

The data that support the findings of this study are included in the article. Further inquiries can be directed at the corresponding author.
